# Spatial statistical learning of task relevance, rather than stimulus prevalence, improves visual working memory recall

**DOI:** 10.3758/s13423-025-02781-8

**Published:** 2026-02-18

**Authors:** Luzi Xu, Andre Sahakian, Stefan Van der Stigchel, Chris L. E. Paffen, Surya Gayet

**Affiliations:** Experimental Psychology, Helmholtz Institute, Utrecht University, Heidelberglaan 1, 3584 CS Utrecht, The Netherlands

**Keywords:** Statistical learning, Working memory, Attention, Task relevance, Stimulus prevalence

## Abstract

Our visual environment provides us with more information than we can process. Processing efficiency may be enhanced by leveraging inherent regularities in the environment, such as prioritizing likely target objects over unlikely ones. Although there is evidence that statistical learning improves visual working memory performance, it is unclear whether this improvement occurs through stimulus prevalence (how likely we are to encounter stimuli) or task relevance (how likely we are to use these stimuli). To distinguish between these two hypotheses, we examined whether frequent appearance, or frequent probing of stimuli (or both) improves visual working memory recall. Participants were asked to recall and replicate the orientation of one of two previously presented oriented gratings (Gabors) as precisely as possible. In two experiments, we manipulated (1) stimulus prevalence by presenting Gabors more frequently on one side (either left or right), and (2) task relevance by probing Gabors more frequently on one side (either left or right). We found comparable orientation recall performance for stimuli appearing at probable versus improbable locations, suggesting that regularities in stimulus prevalence do not improve memory recall. Contrastingly, we found better recall for stimuli appearing at locations that were more (versus less) likely to be probed. Specifically, task relevance enhanced recall performance, both by reducing the number of categorical errors and by increasing fine-grained recall precision. These findings demonstrate that statistical learning of task relevance, but not of stimulus prevalence, enhances visual working memory recall performance.

**Supplementary Information:**

The online version contains supplementary material available at 10.3758/s13423-025-02781-8.

## Introduction

Human beings are proficient in extracting patterns and structures embedded in visual environments. For example, when searching for a book, we typically prioritize probable locations like tabletops and shelves over less probable locations like a chair or the floor. This ability, known as statistical learning, is fundamental to a wide range of both basic and higher-level cognitive processes (for reviews, see Bogaerts et al., 2020; Sherman et al., 2020). Over the last decade, numerous studies on statistical learning have primarily focused on its role in visual attention (for a review, see Theeuwes et al., 2022). Several studies have suggested that visual working memory, a mechanism that is closely related to visual attention (Chun, 2011; Downing, 2000; van Ede & Nobre, 2023) and is essential for maintaining and manipulating visual information, also benefits from statistical learning (Brady et al., 2009; Haines et al., 2023; Umemoto et al., 2010; Won & Leber, 2017). It is surprising, however, that far less is known about which aspects of statistical learning benefit visual working memory performance. It is conceivable that better memory recall for a certain coffee packaging, for example, could result from our frequent behavioral interactions with the product (e.g., regularly searching for the product in the supermarket), but it could also result from being visually exposed to it more often (e.g., regularly seeing it on television). Previous studies on statistical learning often confound these two factors (e.g., Jiang et al., 2013; Umemoto et al., 2010; van Moorselaar & Theeuwes, 2023; Xu et al., 2024), making a probable (versus improbable) stimulus *displayed* more frequently and at the same time *probed* more frequently. It is thus unclear whether stimulus prevalence, task relevance, or both, contribute to the effects of statistical learning on visual working memory.

The hypothesis that statistical learning of stimulus prevalence may enhance visual working memory recall (termed as *the stimulus prevalence hypothesis*) follows from the prevailing theory that statistical learning operates through mere visual exposure implicitly (Aslin, 2017; Fiser & Aslin, 2001), without the involvement of top-down processes driven by explicit goals (i.e., task relevance). According to this theory, frequent visual exposure to probable stimuli should be a critical factor in how statistical learning influences visual working memory. Furthermore, a large body of studies illustrates that memory and related processes (such as attention) benefit from mere frequent exposure to visual stimuli. Stimulus repetition has been shown to lead to quicker target recognition (Van Strien et al., 2005), enhanced attentional processing (Chun & Jiang, 1998), more efficient working memory encoding (Xu et al., 2025), and larger memory capacity (Brady et al., 2009).

On the other hand, there is also evidence supporting the hypothesis that statistical learning facilitates visual working memory in a strategical manner—through task relevance (termed as *the task relevance hypothesis*). In support of this view, a study using a change-detection task found that the repetition of visual arrays alone does not enhance memory performance; instead, memory improvement occurs only when the repetition is linked to task relevance—for instance, when a repeated visual array is associated with a specific probe location where a change frequently occurs (Olson et al., 2005). Consistently, some studies found that frequent probing of an item can lead to better memory recall (Won & Leber, 2017) and faster retrieval of that item (Haines et al., 2023). Theories on visual attention also provide support for *the task relevance hypothesis*: task relevance is considered as a key factor in determining attentional priority, with task-relevant stimuli typically gaining more attentional resources than task-irrelevant stimuli. Since attention gates visual working memory, task-relevant stimuli may gain priority in visual working memory because they receive more attentional resources (Chun, 2011; Downing, 2000; van Ede & Nobre, 2023).

The present study was conducted to investigate whether statistical learning improves visual working memory performance through (1) stimulus prevalence (i.e., learning that stimuli *appear* more frequently at particular locations), (2) task relevance (i.e., learning that stimuli *are probed* more frequently at particular locations), or both. In Experiment 1, we manipulated the prevalence of stimuli at different locations, by presenting to-be-remembered stimuli four times more frequently on one side than the other. In this experiment, the probability of a stimulus being probed on either side was kept constant, thus keeping task relevance equal. In contrast, in Experiment 2, we varied task relevance, by probing stimuli appearing on one side four times more frequently than stimuli appearing on the other side. In this experiment, to-be-remembered stimuli were presented equally often on both sides, thus keeping stimulus prevalence equal. If statistical learning affects visual working memory recall performance—either through stimulus prevalence (Experiment 1) or through task relevance (Experiment 2)—we would observe differential visual working memory recall performance for stimuli in high-probability and low-probability conditions.

Notably, the effects of statistical learning on visual working memory could entail the reduction of *categorical* errors (i.e., failure to memorize items, which may lead to reporting a vertical-oriented Gabor as being horizontal) and/or the increase of *fine-grained* precision (i.e., reduced variances in the recall of memorized items). In fact, previous studies have found statistical learning effects to reduce *categorical* errors (Umemoto et al., 2010; Won & Leber, 2017), but not to increase *fine-grained* precision (Umemoto et al., 2010). Specifically, Umemoto et al. (2010) found that higher detection accuracy for probable locations was present only for *categorical* changes (e.g., a circle changing into a square) but not for *fine-grained* changes (e.g., parallel lines changing into intersecting lines within a shape). The forced choice task that they used, however, only provided *categorical* options, and may therefore not have allowed to establish differences in *fine-grained* recall performance. To address this issue, we employed a continuous report task where participants used a 360° rotating response wheel to reproduce orientation of Gabor patches as precisely as possible. The continuous data we obtained from this task allowed us to distinguish between categorical errors (i.e., large errors indicating that stimuli are not memorized) and fine-grained precision (i.e., minor deviations from correct answers in the recall of memorized stimuli, after excluding categorical errors).

## Experiment 1

In Experiment 1, we tested whether statistical regularities in stimulus prevalence alone influence visual working memory recall performance. To this aim, 80% of the stimuli appeared on one side of the screen (either left or right, varied between participants), with the remaining 20% of stimuli appearing on the opposite side. Any stimulus had a 50% chance of being probed upon presentation, irrespective of whether it was presented on the left or right of fixation. Thus, task relevance was equated between the left and right sides of the display, so that no statistical learning of task relevance could occur.

### Method

#### Participants

Twenty-four participants (12 women and 12 men, mean age = 26.25 years, *SD* = 5.36) were recruited via Prolific (www.prolific.co) and received monetary reward (7.5 GBP per hour). As there were no prior comparable studies, we based the current sample size on prior experience and practical constraints (i.e., financial compensation for participants). Importantly, the use of Bayesian statistics allows us to distinguish between null-effects and experimental insensitivity. All participants self-reported (corrected to) normal vision, indicated to be fluent in English (as the instructions were provided in English), and had an approval rate of 95% or higher in Prolific. All experiments and procedures were approved by the Ethics Committee of the university.

#### Apparatus, procedure, and stimuli

The experiment was programmed in the code editor Visual Studio Code (Version 1.75; https://code.visualstudio.com) using the JavaScript libraries jsPsych (Version 7; de Leeuw, 2015) and Jspsychophysics (Kuroki, 2021). The task was implemented on the web service Gorilla (https://app.gorilla.sc). We required participants to use a laptop or desktop computer along with a computer mouse for the experiment. Data analysis was performed in MATLAB (R2021a; The MathWorks, Natick, MA) and JASP (Version 0.18.1; Love et al., 2019).

**Fig. 1:**
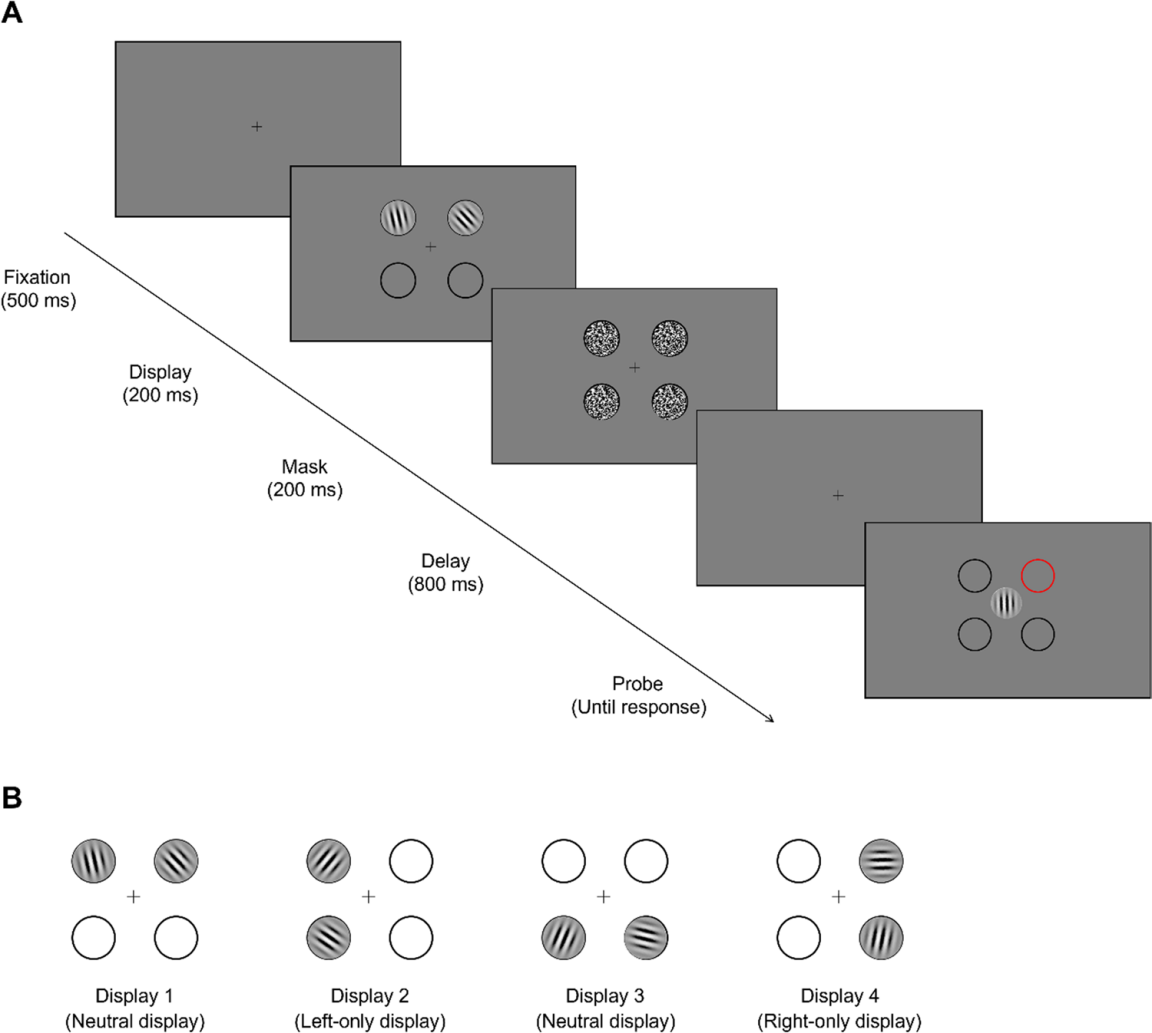
**A** Schematic illustration of a trial from Experiments 1 and 2. Participants began each trial with a central fixation (500 ms), followed by a visual display of two Gabors (200 ms) with random orientations appearing in two of four placeholders. Subsequently, backward masks (200 ms) appeared at the positions of all four placeholders to reduce afterimages. After a delay of 800 ms, one of the placeholders was highlighted in red, instructing participants to reproduce the orientation of the Gabor that was previously presented at that location, by moving the mouse horizontally to rotate a centrally presented response Gabor (without any time constraints). In Experiment 1, stimuli appeared four times more frequently on one side (either left or right), while stimuli on either side were equally likely to be probed for reproduction. In Experiment 2, stimuli appeared equally frequently on the left and right sides, but stimuli on one side (either left or right) were four times more likely to be probed for reproduction. **B** The four different layouts used as memory displays in Experiments 1 and 2 (the two orientations were randomly determined on every trial). (Color figure online)

In this experiment, there were 25 practice trials and 150 formal trials that were divided into 5 blocks of 30 trials each. Each trial (depicted in Fig. 1A) started with the presentation of a central fixation cross for 500 ms. Participants were instructed to fixate the central fixation throughout the trial. Then, the memory display was presented, consisting of two Gabor patches presented for 200 ms within two out of four possible locations (upper left, upper right, lower left, and lower right of fixation). Immediately after the Gabor patches disappeared, Gaussian noise masks appeared for 200 ms within all four placeholders to interfere with iconic memory and reduce afterimages. Following a delay of 800 ms, the outline of one of the (now empty) placeholders was highlighted in red, and a new (horizontally oriented) Gabor patch was presented at fixation. Participants were instructed to reproduce the orientation of one of the two Gabors that were previously presented. Either of the two presented Gabors was equally likely to be probed (i.e., 50% chance) irrespective of the position in the memory display. The location where the probed Gabor was previously presented was highlighted by a red placeholder. Participants used the mouse to rotate the orientation of the central Gabor to replicate the orientation of the probed Gabor: moving the mouse left or right rotated the central Gabor counterclockwise or clockwise, respectively. Participants could then fix the orientation of the Gabor by clicking the left mouse button, after which participants were presented with two options, “register” or “adjust.” Clicking the “adjust” option enabled them to readjust the orientation of the Gabor, while choosing “register” finalized their response for that trial and initiated the next trial. Participants were instructed to replicate the probed Gabor as precisely as possible, with no time constraints. After each block, participants received feedback on their performance (average angular deviation of their registered responses relative to the true stimulus orientations) and got the opportunity to take a short break. At the end of the experiment, to test whether participants were aware of the statistical regularities, we asked participants to indicate on which side of fixation (left or right) they thought stimuli had appeared most frequently. They were asked to also rate their confidence regarding this question on a scale from 1 to 7.

In the memory display, two target stimuli appeared on two out of four possible locations, demarcated by circular placeholders: the upper left, upper right, lower left, and lower right relative to fixation. The two stimuli in a memory display were always presented in horizontally or vertically adjacent placeholders. As is shown in Fig. 1B, this resulted in four possible layouts of the memory display: (1) upper left and upper right (“Display 1”), (2) upper left and lower left (“Display 2”), (3) lower left and lower right (“Display 3”), and (4) upper right and lower right (“Display 4”). To manipulate stimulus prevalence, we varied the frequency of the four display configurations such that Gabors appeared more often on one side of the screen (left or right) across all trials, regardless of the specific display type. Specifically, in 80% of trials, Gabors were presented on the high-probability side (e.g., left), while the remaining 20% of trials had Gabors on the low-probability side (e.g., right). This bias in the ratio of left versus right sides was manipulated across trials, by varying the prevalence of the four displays (Display 1–4). For example, when left was chosen as the probable side, Display 1 and 3 appeared on 16% of all trials each, Display 2 appeared on 64% of all trials, and Display 4 appeared on 4% of all trials. Crucially, in this Experiment, whether a Gabor was presented on the right side or the left side did not predict at all which Gabor was going to be probed during the recall task. Whether the left or right side was more prevalent was counterbalanced across participants. Target stimuli were Gabor patches (sine-wave gratings with a Gaussian envelope) with a standard deviation of 12 pixels, a frequency of 0.1 cycles per pixel, and a fixed phase of 0. On each trial, the orientations of both Gabor patches were randomly selected (range: 0–360°). Thus, there was no manipulation of statistical regularities of the orientations; all frequency manipulations pertained to the stimulus locations. The masks consisted of white noise masks with a Gaussian envelope. On each trial, four masks were randomly drawn without replacement from a set of 40 pregenerated masks.

In an attempt to maximally equate the stimulus sizes for different participants using different displays, we implemented a calibration procedure, before the main experiment. Participants were instructed to place a credit card or other standard-sized card (typically 8.56-cm wide) on the screen, within a designated rectangle. They then adjusted the size of the rectangle using the mouse, to match the dimensions of the card. After calibration, the radius of the Gabor patches and the inner radius of each place holder was approximately 1.1 cm, and the distance between the center of each place holder and fixation (at the center of the screen) was approximately 3.2 cm.

### Data analysis

We used the circular standard deviation (CSD) of the response errors to assess the recall performance of visual working memory. CSD is often used for tasks involving the recall or reproduction of circular features (e.g., Bays, 2016), such as the reproduction of Gabor orientations in our study. It measures the dispersion of response errors, akin to the traditional standard deviation, while taking into account the circular nature of angular data. In the current study, the calculation of CSD involves computing angular differences between each response and the correct orientation, wrapping them within the [− 90°, 90°] range, squaring them, averaging the result, and then taking the square root. As a result, lower CSD values indicate better performance in orientation reproduction.

For all statistical analyses using Bayesian statistics, we used the JASP software with the default priors (for ANOVAs: fixed effects ${r}_{scale}$ = 0.5, random effects ${r}_{scale}$ = 1, covariates ${r}_{scale}$ = 0.354; for *t* tests: a Cauchy distribution with a scale parameter of 0.707) and consistently setting the seed value to 1 for reproducibility (Love et al., 2019).

A directional Bayesian paired-samples *t*-test was conducted to test the main hypothesis of Experiment 1 that statistical learning of stimulus prevalence leads to better working memory recall (i.e., lower CSD values) in the high-probability versus the low-probability condition. The *Bayes Factor* (directional test: ${{\boldsymbol{B}}{\boldsymbol{F}}}_{-0}$; bi-directional test: ${{\boldsymbol{B}}{\boldsymbol{F}}}_{10}$) obtained from these two analyses indicate the degree of evidence for *the alternative hypothesis* (i.e., the two conditions differ). Following convention, we interpret values between 1 and 3 (or between 1 and 1/3) representing inconclusive evidence, values between 3 and 10 (or between 1/3 and 1/10) indicating substantial evidence, values between 10 and 30 (or between 1/10 and 1/30) representing strong evidence, and values larger than 30 (or smaller than 1/30) representing very strong evidence (Kass & Raftery, 1995).

In the analysis described above, we included all data when calculating the circular standard deviation (CSD). This provides an overall measure of recall performance. Next, we set out to dissociate the potential effects of statistical learning on fine-grained recall precision and on coarse-scale categorical errors. Importantly, we wanted to avoid relying on an arbitrary criterion to distinguish between fine-grained recall errors and categorical recall errors (e.g., labeling response errors above 45° as categorical errors). To do so, we tested for the effect of statistical learning on recall performance in two different sets of analyses, focusing either on fine-grained precision or categorical errors, while iterating through an exhaustive range of criteria for defining “categorical errors.”

First, to isolate fine-grained recall precision, we conducted directional Bayesian *t* tests, testing whether the CSD of the response error was smaller in the high-probability condition than the low probability condition, excluding all trials above a certain criterion (i.e., “categorical errors”). We systematically iterated through multiple exclusion criteria, covering the full range of possible response errors (0°, 90°) in steps of 1°. Accordingly, a criterion of 0° would exclude all trials from analysis, a threshold of 45° would include only trials with a response error below 45°, and a criterion of 90° would include all trials from analysis (akin to the main analyses, described above). If statistical learning improves fine-grained recall precision, we would see evidence (e.g., ${{\boldsymbol{B}}{\boldsymbol{F}}}_{-0}$ > 3) that the CSD is smaller in the high-probability condition than in the low-probability condition, after excluding trials with “categorical errors” (e.g., errors of 45° or more).

Second, to isolate categorical recall errors, we again iterated through all possible response error magnitudes (0°, 90°, in steps of 1°). In this case, however, we computed the proportion of trials with a response error exceeding this criterion. In this case, a criterion of 0° labels all trials as categorical errors (so the proportion of “categorical” errors is 1), and a criterion of 90° labels none of the trials as categorical errors (so the proportion of “categorical errors” is 0). For most values in between (e.g., 45°), there is a proportion of trials with “categorical errors” (in this example, errors of 45° or more). If statistical learning reduces the number of categorical errors, we will see evidence (e.g., ${{\boldsymbol{B}}{\boldsymbol{F}}}_{-0}$ > 3) for a smaller proportion of ‘categorical errors’ in the high-probability condition, compared to the low-probability condition (e.g., when labeling trials as “categorical errors” when they exceed 45°). Note that categorical/coarse-scale recall errors are measured as a proportion of trials (rather than using the CSD), because the exact magnitude of a categorical error is, by definition, irrelevant.

Third, we assessed whether any observed difference in memory performance between high- and low-probability conditions reflects intertrial priming effects (i.e., better recall performance when the same location is probed on two consecutive trials) rather than statistical learning effects (i.e., better recall performance when high-probability locations are probed, independently of the location that was probed on the previous trial). Because stimuli in high-probability locations are inherently more likely to be probed on two consecutive trials than stimuli in low probability locations, we wanted to ensure that a potential effect of stimulus prevalence on memory performance was not caused by mere repetition of the probe on two consecutive trials. To examine whether such priming (or: “N − 1”) effects indeed affect working memory performance, we conducted a Bayesian paired-samples *t* test (repetition: repeat vs. change) to compare participants’ performance when the probed item was at exactly *the same location* (i.e., “repeat”) as in the previous trial, to performance when the probed item was at *the other location* (i.e., “change”) of the same side compared to the previous trial (irrespective of whether it is a high-probability or low-probability side). The comparison of change versus repeat trials within the same probability-side (rather than between probability-sides) enables us to examine such priming effects independently of a potential effect of stimulus prevalence (in Experiment 1) or task relevance (Experiment 2). To test for these priming effects, a Bayesian paired-samples *t* test was conducted to test the effects of Repetition (repeat vs. change). Note that these analyses of intertrial priming effects test an alternative explanation for a difference in recall performance between high and low probability conditions, and are therefore redundant when no such difference is observed to begin with. Thus, we only report on these analyses when a difference in recall performance between high and low probability conditions is observed.

Finally, we conducted analysis on participants’ awareness of the manipulated statistical regularities. These analyses address two main questions. The first and main question concerns whether participants have different levels of awareness in Experiments 1 and 2. This question is crucial to the present study, as differences in awareness levels between Experiments 1 and 2 could drive potential differences in behavioral outcomes between these experiments (instead of the intended manipulation of stimulus prevalence versus task relevance). The second, and more exploratory question, concerns whether differences in awareness about the statistical regularities predict differences in memory performance between high-probability and low-probability conditions (see Supplementary Materials S3).

For each analysis, we employed two complementary approaches to assess awareness levels: (1) a combined measure integrating objective accuracy in the awareness test with subjective confidence ratings (signed confidence weighting), and (2) a purely objective accuracy measure that does not include subjective confidence judgements. In the first approach, we incorporated the results of the subjective confidence ratings with the objective forced-choice reports for a comprehensive assessment of awareness: if they correctly reported the high-probability (displayed or probed) side, their confidence score was recorded as a positive number; if incorrect, it was recorded as a negative number. This value was used as a proxy for their subjective awareness of statistical regularities. In the second approach, we only incorporate objective forced-choice reports (excluding subjective confidence ratings) to ensure that the inclusion of subjective confidence reports did not water down the relation between awareness levels and task performance.

To address the first and main question, we compared awareness across experiments using a Bayesian Mann–Whitney *U* test for composite scores (where correct reports of the high-probability side were coded as positive values weighted by confidence ratings, and incorrect reports were coded as negative values), and a Bayesian contingency table analysis for the raw forced-choice responses (correct side vs. incorrect side vs. no-preference). As mentioned above, the former approach captures subjective awareness strength through signed confidence weighting, while the latter provides an objective accuracy measure unaffected by subjective confidence judgements.

### Results

#### Statistical learning effect

**Fig. 2:**
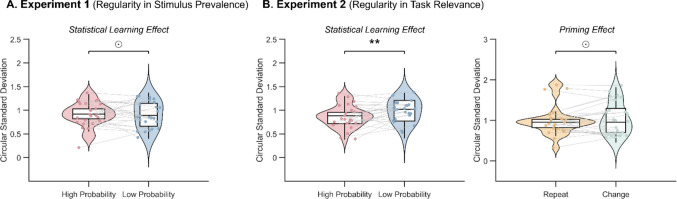
Results of Experiments 1 and 2. **A** and the left panel of **B** display comparisons of circular standard deviations (CSD) between high-probability and low-probability locations. The right panel of **B** displays comparisons of CSD between repeat trials (i.e., items appearing at the same location as in the previous trial) and change trials (i.e., items appearing at a different location from the previous trial). **Bayes factor > 10, Ø: 1/3 < Bayes factor < 3, ⊙: Bayes factor < 1/3. (Color figure online)

Bayesian directional *t*-tests provide substantial evidence (${{\boldsymbol{B}}{\boldsymbol{F}}}_{-0}$ = 0.15) against an effect of stimulus prevalence on recall performance (high probability: CSD = 0.93, *SD* = 0.24; low probability: CSD = 0.90, *SD* = 0.29), as shown in Fig. 2A. This result indicates that statistical regularities in stimulus prevalence alone do not influence visual working memory recall performance; stimuli that appear at more likely locations are not memorized better.

**Fig. 3:**
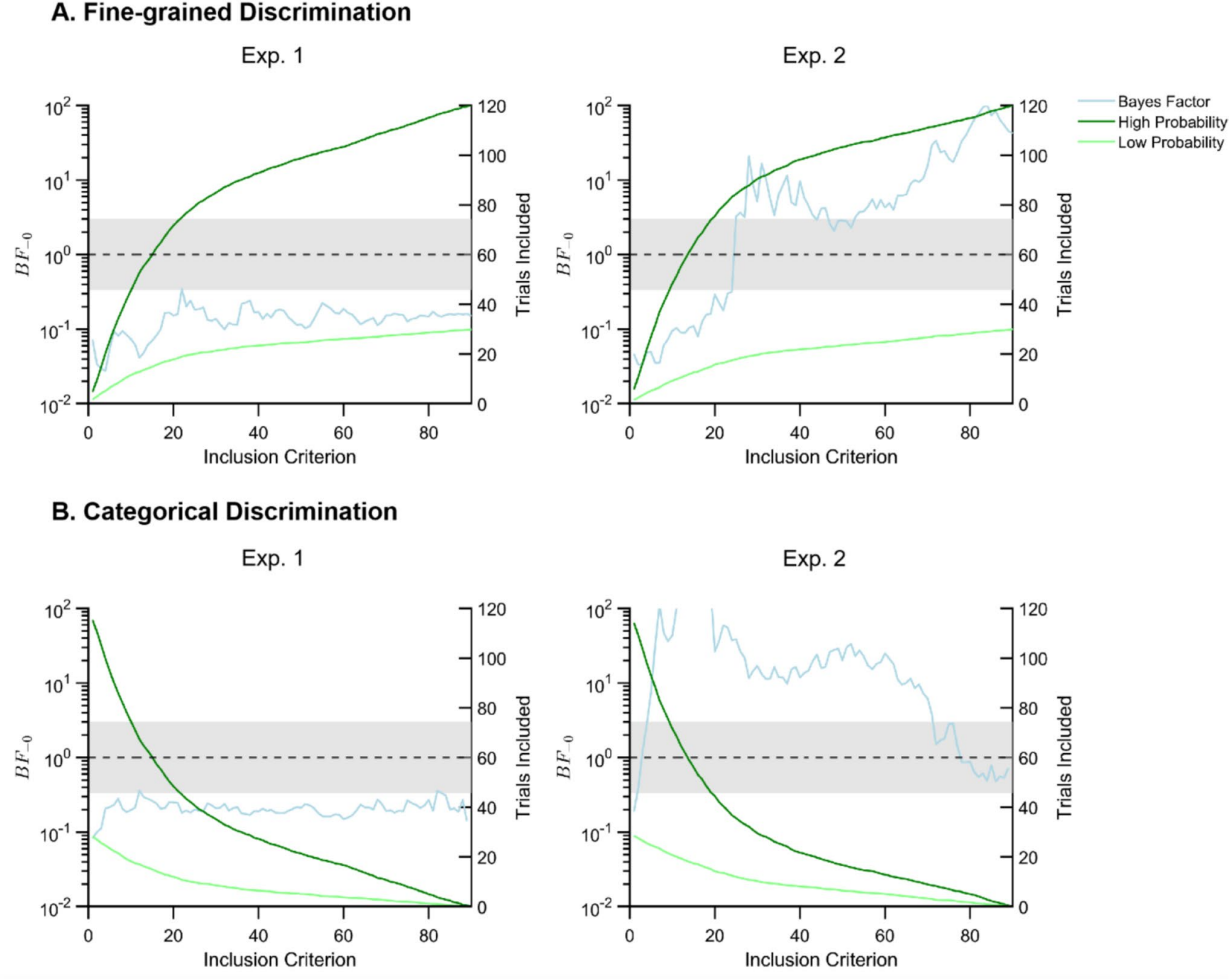
The Bayes factors (left *y*-axis) for the difference in memory recall performance between high- and low-probability locations, for an exhaustive range of inclusion criteria (on the x-axis), and for two outcome metrics (on Panel **A** and **B**). Panel **A** depicts the difference in CSD between probability conditions, for all trials up to the response error depicted on the *x*-axis (e.g., at a value of 45, the CSD is computed for all trials with an absolute error below 45). Panel **B** depicts the difference in error rate between probability conditions, given the threshold value depicted on the* x*-axis (e.g., at a value of 45, the error rate reflects the proportion of trials with an error above 45). The blue line shows the Bayes factors for the directional *t* test, testing whether recall performance is better (lower CSD or error rate) for high-probability locations than for low-probability locations. The dark green curves depict the number of included trials in the high-probability condition, and the light green curves depict the number of trials in the low-probability condition (right *y*-axis). The shaded areas cover [$\frac{1}{3} \ge {{\boldsymbol{B}}{\boldsymbol{F}}}_{-0} \le 3$], so that values above this area provide evidence for better recall performance in the high-probability condition compared to the low-probability condition, and values below this area provide evidence against such a difference. Values within the shaded area reflect insufficient evidence for either hypothesis. (Color figure online)

Next, we specifically tested for differences in fine-grained recall precision, by excluding categorical errors (regardless of the definition of a “categorical error”). As shown in Fig. 3A (left), CSD at high-probability locations was not lower than at low-probability locations (${{\boldsymbol{B}}{\boldsymbol{F}}}_{-0}$ < 1/3).

When specifically investigating categorical errors, we found that the proportion of categorical errors was not lower at high-probability locations than at low-probability locations (${{\boldsymbol{B}}{\boldsymbol{F}}}_{-0}$ < 1/3), regardless of the definition of a “categorical error.” Thus, statistical learning of stimulus prevalence does not improve visual working memory recall, neither by reducing categorical errors nor by increasing fine-grained precision.

#### Awareness of statistical regularities

Among 24 participants, 17 correctly identified the high-probability location (including 10 with above-median confidence), two chose the wrong side, and five reported no difference.

### Interim discussion

In Experiment 1, we showed that presenting items more frequently at a certain location than at another location (given equal probe probability) did not improve memory recall, neither through a reduction in categorical errors nor through an increase in recall precision for stimuli appearing at high-probability locations. The absence of an effect was confirmed by Bayesian evidence for the null. We considered the possibility that the presentation duration of the memory display (i.e., 200 ms) might have led to near-floor performance, thus dampening a potential effect of statistical learning on recall performance. Therefore, in a supplementary experiment, we prolonged the presentation time of the memory display to 450 ms and confirmed that statistical regularity in stimulus prevalence does not improve visual working memory recall (see Supplementary Materials S1).

## Experiment 2

Next, we set out to test whether statistical learning of task relevance (i.e., the likelihood of a stimulus being probed) does affect visual working memory recall performance. To manipulate task relevance, we probed stimuli appearing on one side of the screen (either left or right) on 80% of trials, thus probing stimuli on the other side of the screen only on 20% of trials. In this experiment, memory items appeared on both sides of the display with equal probability.

### Methods

#### Participants

A new group of twenty-four participants (17 women and 7 men, mean age = 28.54 years, *SD* = 4.91) were recruited via Prolific (www.prolific.co). Because learned probabilities cannot be ‘unlearned’ immediately, manipulations of statistical regularities inevitably lead to carry-over effects into subsequent experimental blocks. To prevent carry-over effects between manipulations of statistical regularities in our study (i.e., stimulus prevalence in Experiment 1 vs. task relevance in Experiment 2), we employed a between-subjects design with independent participant groups. The alternative approach of counterbalancing the order of conditions (i.e., experiments) across participants would not solve this issue, as it would (1) add another between-subject condition (namely, block order), and (2) introduce complex and unpredictable interaction terms between block order and probability manipulation. The between-subjects design thus provides the cleanest measurement of different statistical manipulations (in otherwise identical tasks), despite its reduced statistical sensitivity compared with within-subjects alternatives.

#### Apparatus, stimuli, and procedure

The apparatus, stimuli, and experimental procedure in Experiment 2 were identical to Experiment 1, except that (1) stimuli appeared equally frequently on left and right sides of the screen (50% each) and that (2) stimuli were probed more frequently when they appeared on either the left side or the right side of the screen (80%) compared with the opposite side (20%). To achieve this ratio, we adjusted the occurrence of different displays, making neutral displays (see Displays 1 and 3 in Fig. 1) more frequent than others; Display 1 and 3 appeared on 38% of all trials each, while Display 2 and 4 appeared on 12% of all trials each. This differential prevalence of displays allowed us to obtain the 80–20% ratio in the manipulation of probe probability. Which side was probed more often was varied (and counterbalanced) between participants.

### Results

#### Statistical learning effect

A Bayesian directional *t*-test provided strong evidence (${{\boldsymbol{B}}{\boldsymbol{F}}}_{-0}$ = 42.36) that recall performance was better for items that were more likely to be probed (high probability: CSD = 0.87, *SD* = 0.23; low probability: CSD = 0.99, *SD* = 0.25), see Fig. 2B (left). This effect replicated (${{\boldsymbol{B}}{\boldsymbol{F}}}_{-0}$ = 65.84) when analyzing only trials with neutral displays, thereby confirming that recall performance was indeed better for high-probe probability *locations*, rather than for high-probability *displays* (see Supplementary Materials 2).

Next, we tested for differences in recall precision. As shown in Fig. 3A (right), the CSD was lower in the high-probability than the low-probability condition, when excluding categorical errors (${{\boldsymbol{B}}{\boldsymbol{F}}}_{-0}$ > 3). This was the case for a wide range of sensible criteria for defining errors as “categorical,” ranging from very strict (25˚) to very loose definitions of a categorical error (45˚ and higher). This finding demonstrates that statistical learning of task relevance influences the fine-grained recall precision of visual working memory.

Moreover, the proportion of categorical errors was lower in the high-probability condition than in the low-probability condition (${{\boldsymbol{B}}{\boldsymbol{F}}}_{-0}$ > 3), as shown in Fig. 3B (right). This was the case for a wide range of criteria for defining errors as “categorical,” ranging from very strict (5˚) to very loose definitions of a categorical error (up to 70˚). This shows that statistical learning of task relevance reduces the amount of categorical errors.

#### Priming effect

There was substantial evidence against (${{\boldsymbol{B}}{\boldsymbol{F}}}_{10}$ = 0.28) an effect of intertrial priming on recall precision (repeat: CSD = 1.00, **SD** = 0.37; change: CSD = 1.03, *SD* = 0.39), see Fig. 2B right. This confirms that the enhanced recall performance (in the high compared to low-probability condition) is caused by statistical learning of probe probability, and not by the larger prevalence of repeat trials in the high-probability condition.

#### Awareness of statistical regularities

Among 24 participants, 14 correctly identified the high-probability location (including seven with above-median confidence), two chose the wrong side, and eight reported no difference.

#### Comparison between Experiments 1 and 2

To test whether stimulus prevalence (Experiment 1) and task relevance (Experiment 2) differentially impact recall performance, we compared the statistical learning effects (i.e., the difference in recall precision between high and low probability sides) between the two experiments. A Bayesian independent-samples *t* test provided substantial evidence for the difference between the statistical learning effects in the two experiments (Experiment 1: ΔCSD = 0.02, SD = 0.23; Experiment 2: ΔCSD =  − 0.12, *SD* = 0.16), ${{\boldsymbol{B}}{\boldsymbol{F}}}_{10}$ = 3.17.

Next, we tested whether the difference in statistical learning effects on recall performance between the two experiments may have resulted from differences in participants’ awareness of the statistical regularities. A Bayesian contingency table analysis comparing Experiments 1 (*n* = 24) and 2 (*n* = 24) found ​substantial evidence for the null hypothesis of identical response distributions across choices of high-probability side, low-probability side, and no difference (${{\boldsymbol{B}}{\boldsymbol{F}}}_{10}$= 0.18, Dirichlet prior *α* = 1). Confirming these findings, results of a Bayesian Mann–Whitney (i.e., independent-samples nonparametric) *t* test showed substantial evidence *against* a difference in awareness of statistical regularities between experiments (Experiment 1: mean = 1.25, *SD* = 3.50; Experiment 2: mean = 0.75, *SD* = 3.43), ${{\boldsymbol{B}}{\boldsymbol{F}}}_{10}$ = 0.31.

Thus, statistical learning of task relevance influences recall precision more than statistical learning of stimulus prevalence does, and this is not caused by a difference in awareness of the statistical regularities.

### Interim discussion

In Experiment 2, we found that statistical learning of task relevance affects visual working memory recall performance, even when stimulus prevalence is equated. This effect persisted when excluding categorical errors (even with definitions of categorical errors as strict as 25°), demonstrating that statistical regularities affect the fine-grained recall precision of visual working memory representations. The effect of statistical regularities on recall performance was overall more pronounced for participants who were more aware of the statistical regularities (Supplementary Materials 3), which may indicate that deliberate strategies play a role in the present findings. Furthermore, we observed no evidence for intertrial priming effects; probing a stimulus at the exact same location on two consecutive trials (which inevitably occurs more often in the high-probability condition than the low-probability condition) does not improve recall performance. Thus, the effects of task relevance identified here cannot be attributed to inter-trial priming effects, and should therefore be attributed to statistical learning, that is, the learning of the overall distributional probability of task-relevant items.

## General discussion

In this study, we investigated whether statistical learning enhances visual working memory recall through learning of *stimulus prevalence* (e.g., stimuli appeared more frequently on certain locations), *task relevance* (e.g., stimuli were probed more frequently on certain locations), or both. Experiment 1 (and the Supplementary Experiment) showed that recall performance for stimuli appearing at more probable locations was not better than those appearing at less probable locations, neither reducing *categorical* errors (i.e., the likelihood of remembering an item) nor increasing *fine-grained* precision (after excluding categorical errors). In contrast, Experiment 2 showed that regularities in *task relevance* did improve visual working memory recall, resulting in a smaller proportion of categorical errors and an increase in recall precision for stimuli that were more likely to be probed compared with those that were less likely to be probed. Importantly, Experiments 1 and 2 were directly comparable, because participants performed the same task on the same stimuli, and the statistical regularities were equal in objective strength (a 4:1 ratio between high and low-probability conditions) as well as subjective strength (i.e., no difference in the awareness measures between experiments). Together, these findings demonstrate that statistical learning improves visual working memory recall through task relevance, but not stimulus prevalence.

The lack of benefit (Experiment 1)—and even potential impairment (Supplementary Experiment)—for stimuli appearing at high-prevalence locations may seem counterintuitive, since the high-probability location was more likely to contain stimuli, and was therefore also more likely to contain the task-relevant target stimulus—theoretically, even if participants would only attend the high-probability side throughout the entire experiment, they would have an 80% probability of seeing the probed item (the same ratio as Experiment 2). This lack of recall benefit at likely stimulus locations is consistent, however, with previous work showing that simply repeating the same multi-item display throughout an experiment does not increase change-detection accuracy; change-detection accuracy was only shown to improve for repeated displays when the same location was probed (Olson et al., 2005). Thus, visual repetition of a display enhances recall performance only when the repeated display was associated with a task-relevant location (i.e., a repeated probe location). In Experiment 1 and the Supplementary Experiment, we purposefully dissociated stimulus prevalence from task relevance. Accordingly, despite the high-probability location inevitably containing the target stimuli more often, it also contained more distractor stimuli, which participants may have learned to ignore or suppress (Wang & Theeuwes, 2018).

In contrast, when manipulating how often items in a particular location would be probed (i.e., task relevance) while keeping stimulus prevalence constant, memory recall performance was substantially higher for stimuli that were probed at high-probability locations. This finding aligns with that of two previous studies showing that repeated probing of the same location or feature facilitates memory recall (Umemoto et al., 2010; Won & Leber, 2017). Moreover, this finding aligns with Zhang and Carlisle (2023), showing that spatial priority maps guide attention only when learned regularities directly match current task goals. Our work, together with that of Zhang and Carlisle (2023), provides converging evidence that task relevance is critical for unlocking statistical learning, while challenging the traditional view put forward in location probability cueing studies (e.g., Jiang, 2018; Jiang et al., 2013) that spatial biases are persistent and independent of current task goals.

Dissociating the effects of task relevance and stimulus prevalence required a design that isolates these factors. Ideally, a full-factorial design would be employed that (1) allows to test this distinction within-subjects, and (2) allows to test possible interactions between these factors. However, simultaneously manipulating task relevance and stimulus prevalence would likely induce global strategic shifts in cognitive control (Koch & Allport, 2006; Rogers & Monsell, 1995). Previous studies showed that mixing different manipulations fundamentally alters the cognitive demands of the entire task and a pervasive state of global cognitive control (Rogers & Monsell, 1995). Contradictory manipulations require participants to maintain attentional sets for the same spatial location (Koch & Allport, 2006), yet task-relevance is a powerful top-down factor that can reconfigure basic attentional processes (Woodman & Luck, 2003). Thus, participants may not maintain two opposing patterns, but instead adopt a single, global strategy to resolve the conflict and simplify the task, such as strategically suppressing the unreliable stimulus prevalence dimension altogether. Future work may develop novel within-subject paradigms to overcome these various challenges, while also offering the possibility to uncover potential interactions between these distinct types of statistical learning.

Another open question pertains to the processing stage at which the observed dissociation (between effects of stimulus prevalence and task relevance) occurs: during statistical learning, the allocation of spatial attention, or working memory maintenance. As outlined above, explicit learning was comparable across conditions, so the differences in recall performance cannot be attributed to differences in learning strength. Given the role of attention in gating visual working memory (Chun, 2011; Downing, 2000; van Ede & Nobre, 2023), it is possible that—from the statistical regularities—participants learned which location to attend prior to stimulus presentation. This strategy is particularly useful in the current task design with brief stimulus presentations (200 ms). But why would statistical learning influence the allocation of (spatial) attention in Experiment 2, but not in Experiment 1, where attending the high probability side at the expense of the low probability side during stimulus encoding would be (objectively) equally beneficial? A third possibility, then, is that attention prioritizes items that are more likely to be probed over items that are less likely to be probed after encoding; that is, during working memory maintenance. It is even possible that some items were prioritized during perceptual encoding (including stimuli at high prevalence locations) but are deprioritized during subsequent maintenance if they are not particularly likely to be probed. This scenario would explain why probing stimuli on one side more frequently enhances memory recall, but displaying stimuli more frequently does not. Future studies incorporating continuous measures of attentional selection (e.g., directional gaze biases, lateralized EEG components, etc.) could provide definitive evidence to dissociate between these possibilities.

Notably, our finding that statistical learning enhanced fine-grained precision (in Experiment 2) contradicts the earlier conclusion by Umemoto et al. (2010) that it only affects categorical errors. Umemoto et al. (2010) used a change-detection (i.e., two-alternative forced-choice) task and found that—at frequently probed locations—change-detection accuracy improved for changes in categorical information (i.e., overall shape of an item) but not for changes in fine-grained information (i.e., small elements within that shape). We attribute this discrepancy to key methodological differences. First, our continuous report task is inherently more sensitive to precision changes than the two-alternative forced-choice task used by Umemoto et al. (2010). Second, unlike their fixed definitions of errors and precision, we employed a parametric approach that exhaustively tests a range of criteria, avoiding arbitrary thresholds and providing a more robust dissociation between categorical and fine-grained errors. Using a more sensitive approach, the present study goes beyond earlier work that statistical learning increases the number of items stored in working memory (Brady et al., 2009; Umemoto et al., 2010), by highlighting that statistical learning can improve the quality (or resolution) of visual working memory content.

Our findings, together with previous studies (Zhang & Carlisle, 2023), prompt a reconsideration of the idea that statistical learning—at large—operates solely as an implicit mechanism through mere exposure (Aslin, 2017), independent of top-down processes driven by explicit goals or task relevance (for a review, see Luck et al., 2021; Theeuwes et al., 2022). Instead, although distributional probabilities may be learned implicitly, their influence on perceptual, mnemonic, and attentional processes may be contingent on the behavioral goals of the observer.

## Supplementary Information

Below is the link to the electronic supplementary material.

Supplementary file1 (DOCX 144 kb)

## Data Availability

All data have been made publicly available at Open Science Framework and can be accessed at https://osf.io/q6sbu/?view_only=0d955063aa82477eab24e55d0a9185ec. The materials used in the study were common visual stimuli (Gabor patches), and the specific parameters have been detailed in the Methods section.

## Code availability

All code used for data analysis is available upon request.
