# Supplementary material for: Spatial statistical learning of task relevance, rather than stimulus prevalence, improves visual working memory recall
Source: Psychon Bull Rev. 2026 Feb 18;33(2):80. doi: 10.3758/s13423-025-02781-8 (PMC12916991; doi:10.3758/s13423-025-02781-8)
Supplement: Supplementary file 1 — Supplementary file1 (DOCX 144 kb) [file 13423_2025_2781_MOESM1_ESM.docx]

**Supplementary Materials for:**

**Spatial statistical learning of task relevance, but not stimulus prevalence, improves working memory recall**

#### Supplementary Materials 1: Supplementary Experiment

In Experiment 1, we observed no significant difference in orientation recall performance between high-probability locations (where stimuli were presented four times more often) and low-probability locations. To ensure the robustness of this finding, we conducted a supplementary experiment with the same stimuli and procedures, but with a longer presentation duration of the memory display. This would provide observers with more time to encode the stimuli, thus reducing the task-difficulty compared to Experiment 1, and thereby possibly increasing the experimental sensitivity to detect a difference in performance between probability conditions.

**Method**

***Participants***

A new group of twenty-four participants (12 women and 12 men, mean age = 29.17, SD = 4.68) were recruited via Prolific (www.prolific.co) as in Experiment 1.

***Apparatus, stimuli & procedure***

The experimental settings were identical to those in Experiment 1, except that the presentation time of the memory display was set to 450 ms (instead of 200 ms in Experiment 1) and that there were fewer practice trials (five practice trials).

**Results**

***Statistical learning effect*.** Results of a directional Bayesian *t*-test provided strong evidence ($\boldsymbol{BF}_{\boldsymbol{-}\boldsymbol{0}}$ = 0.07) against the hypothesis that the circular standard deviation (CSD) of the response error is smaller at high-probability locations (0.86, SD = 0.18) than at low-probability locations (0.76, SD = 0.24). This replicates the key finding of Experiment 1. To explore whether recall performance was actually worse at high-probability locations than at low-probability locations, we further conducted an exploratory bidirectional Bayesian *t* test and found substantial evidence for a higher CSD for high-probability compared to low-probability locations ($\boldsymbol{BF}_{\boldsymbol{1}\boldsymbol{0}}$ = 3.46). While it is unexpected that memory performance was worse for high-probability locations compared to low probability locations, the present results do confirm the findings of Experiment 1 that increasing stimulus prevalence (while keeping task relevance constant) does not *enhance* visual working memory performance. Together, these results indicate that statistical regularities in stimulus prevalence do not improve visual working memory recall performance.

As in Experiment 1, we also conducted directional Bayesian *t* tests to examine the CSD of responses to high- versus low-probability locations with different data inclusion thresholds (see Method of Experiment 1 in the main document for more details). Consistent with Experiment 1, results of Bayesian directional *t* tests consistently showed that, irrespective of inclusion threshold, both CSD (for isolating fine-grained discrimination, see Suppl. Fig.1A) and error rate (for isolating categorical discrimination, see Suppl. Fig.1A) were never lower at high-probability locations than at low-probability locations ($\boldsymbol{BF}_{\boldsymbol{-}\boldsymbol{0}}$ < 1). These analyses provide further confirmation that statistical learning of stimulus prevalence does not improve visual working memory recall, neither at the level of categorical discrimination nor at the level of fine-grained discrimination.


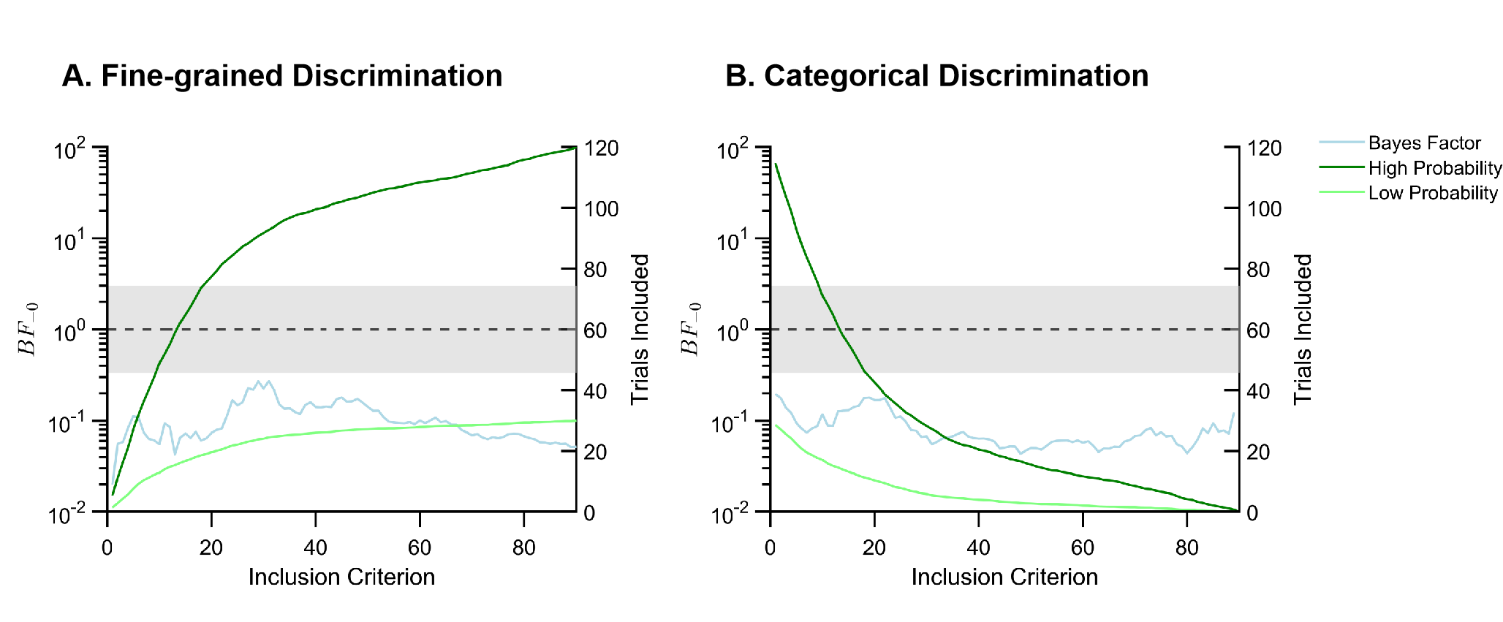


**Suppl. Fig. 1.** The Bayes Factors (left y-axis) for the difference in memory recall performance between high-probability and low-probability locations, for an exhaustive range of inclusion criteria (on the x-axis), and for two outcome metrics (on Panel A and B). Panel A depicts the difference in CSD between probability conditions, for all trials up to the response error depicted on the x-axis (e.g., at a value of 45, the CSD is computed for all trials with an absolute error below 45). Panel B depicts the difference in error rate between probability conditions, given the threshold value depicted on the x-axis (e.g., at a value of 45, the error rate reflects the proportion of trials with an error above 45). The blue line shows the Bayes Factors for the directional *t* test, testing whether recall performance is better (lower CSD or error rate) for high-probability locations than for low-probability locations. The dark green curves depict the number of included trials in the high-probability condition, and the light green curves depict the number of trials in the low-probability condition (right y-axis). The shaded areas cover [$\frac{1}{3} \geq\boldsymbol{BF}_{\boldsymbol{-0}} \leq3$], so that values above this area provide evidence for better recall performance in the high-probability condition compared to the low-probability condition, and values below this area provide evidence against such a difference. Values within the shaded area reflect insufficient evidence for either hypothesis.

***Awareness of statistical regularities.*** Among 24 participants, 12 correctly identified the high-probability location (including 7 with above-median confidence), 5 chose the wrong side, and 7 reported no difference.

#### Supplementary Materials 2: Accounting for display probability

In all experiments, we manipulated the frequencies of different displays (Display 1-4) to vary the probabilities of items being presented or probed on the left versus right sides of fixation. This manipulation, however, inevitably introduced display-based probabilities (i.e., some displays occurring more frequently than others) on top of the intended location-based probabilities (e.g., one side of fixation occurring more frequently than others). In our analysis in the main manuscript, the high and low location probability conditions were also high and low display probability conditions. To isolate the effects of high and low *location* probability from potential effects of *display* probability, we conducted a supplementary analysis. Specifically, we compared memory performance for probable versus improbable sides (left or right) within one type of display; the neutral display, which contains one stimulus on each side of fixation (i.e., Display 1 and 3). By contrasting memory recall performance for high versus low location probability conditions within a display, we eliminated any influence of display probability on the results.

An alternative approach is to compare performance between Display 2 (all high-probability locations) and Display 4 (all low-probability locations). While this comparison might be particularly sensitive to statistical learning effects, this approach cannot distinguish whether learning was based on the spatial location of items or on the recognition and prioritization of the global display configuration itself. In contrast, the within-display comparison for neutral displays (Displays 1 and 3) provides a conclusive test, specifically measuring the effects of spatial (location-based) learning. By holding the global display configuration constant, any performance advantage for the high-probability location within a neutral display can be exclusively attributed to learning tied to the spatial location itself, definitively ruling out display-based learning as an alternative explanation.

**Experiment 1**

Results provided substantial evidence against the difference in CSD between high- and low-probability sides within neutral displays alone, $\boldsymbol{BF}_{\boldsymbol{-0}}$ = 0.17. Consistent with the main analysis, these results indicate that statistical learning of stimulus prevalence does not improve recall performance.

**Experiment 2**

Results provided substantial evidence for a smaller CSD on high- compared to low-probability sides within neutral displays alone, $\boldsymbol{BF}_{\boldsymbol{-0}}$ = 65.84. Consistent with the main analysis, these results indicate that statistical learning of task relevance improves recall performance, leading to better recall for stimuli at frequently probed locations.

**Supplementary Experiment**

Results provided substantial evidence against the difference in CSD between high- and low-probability sides within neutral displays alone, $\boldsymbol{BF}_{\boldsymbol{-0}}$ = 0.14. Consistent with the main analysis, these results indicate that statistical learning of stimulus prevalence does not improve recall performance.

**Conclusion**

These supplementary analyses confirm the main conclusion that statistical learning of task relevance (Experiment 2) but not stimulus prevalence (Experiment 1 and Supplementary Experiment) improves visual working memory recall.

#### Supplementary materials 3: Relation between awareness of the statistical regularities and their impact on recall performance

Here, we explore whether participants’ awareness of the manipulated statistical regularities predict the difference in recall performance between high- and low-probability conditions. As described in the main document (see Methods of Experiment 1), we employed two complementary approaches to assess awareness levels: (1) a combined measure integrating objective accuracy in the awareness test with subjective confidence ratings (signed confidence weighting), and (2) a pure objective accuracy measure that is unaffected by subjective confidence judgements.

For the combined measure incorporating confidence ratings, the awareness ratings are categorical. We therefore conducted a Kendall’s tau Bayesian correlation test between the signed confidence weights and the difference in memory recall performance between probability conditions.

For the objective accuracy measure, we conducted a Bayesian Analysis of Variance (ANOVA), with Group as between-subject factor (comparing “correct guessers”, defined as participants who correctly identified the high-probability side in the awareness test, and “incorrect guessers”, defined as those who either misidentified the low-probability side as high-probability or reported no difference between sides) and Probability as a within-subject factor (high- versus low-probability).

**Experiment 1**

Analyses showed inconclusive evidence for a difference in statistical learning effects on memory recall (i.e., the CSD difference between high- and low-probability locations) between correct and incorrect guessers ($\boldsymbol{BF}_{\boldsymbol{10}}$ = 1.20). Similarly, we found inconclusive evidence for a correlation between awareness of statistical regularities and the CSD difference between high- and low-probability conditions, Kendall’s tau = -0.24, $\boldsymbol{BF}_{\boldsymbol{10}}$ = 0.98.

**Experiment 2**

Analyses showed inconclusive evidence for a difference in statistical learning effects on memory recall between correct and incorrect guessers ($\boldsymbol{BF}_{\boldsymbol{10}}$ = 2.08). However, the analysis incorporating confidence measures provided substantial evidence for a positive correlation between awareness of statistical regularities and the statistical learning effect on recall precision (i.e., the CSD difference between high and low probability conditions), Kendall’s tau = 0.34, $\boldsymbol{BF}_{\boldsymbol{10}}$ = 3.29. Together, these results indicate that awareness of statistical regularities may contribute to the statistical learning effects that we observed.

Note that although participants’ awareness of statistical regularities predict the extent to which statistical learning affects recall performance in Experiment 2, it cannot explain the different results obtained in Experiment 1 (i.e., the absence of statistical learning effect) and 2 (i.e., the presence of statistical learning effect), since awareness levels did not differ between Experiments (see ‘Comparison between Experiments 1 and 2’ in the main manuscript).

**Supplementary Experiment**

We found inconclusive evidence for a difference in the effect of statistical learning on memory recall between correct and incorrect guessers ($\boldsymbol{BF}_{\boldsymbol{10}}$ = 0.41). Similarly, the analysis incorporating confidence measures provided inconclusive evidence for the correlation between participants’ awareness of statistical regularities and the magnitude of the effect of statistical learning on recall performance (i.e., the CSD difference between high- and low-probability conditions), Kendall’s tau = 0.16, $\boldsymbol{BF}_{\boldsymbol{10}}$ = 0.46.

#### Supplementary materials 4: Analysis of response times

Despite responses in our task being non-speeded, we conducted an exploratory analysis of response times (RTs) between high- and low-probability locations to ensure a comprehensive understanding of the data.

**Experiment 1**

Results of a directional Bayesian *t*-test provided substantial evidence ($\boldsymbol{BF}_{\boldsymbol{-0}}$ = 0.33) against the effect of statistical learning on RTs (high-probability locations: 3.41, SD = 1.85; low-probability locations: 3.47, SD = 1.94).

**Experiment 2**

Results provided inconclusive evidence ($\boldsymbol{BF}_{\boldsymbol{-0}}$ = 0.33) against the effect of statistical learning on RTs (high-probability locations: 3.62, SD = 1.42; low-probability locations: 3.89, SD = 3.43).

**Experiment 3**

Results provided substantial evidence ($\boldsymbol{BF}_{\boldsymbol{-0}}$ = 0.20) against the effect of statistical learning on RTs (high-probability locations: 4.28, SD = 2.89; low-probability locations: 4.25, SD = 2.46).
